# Sleep problems and emotional and behavioral problems among adolescents: examining independent types, cumulative scores, and latent patterns

**DOI:** 10.1186/s13034-026-01104-6

**Published:** 2026-06-03

**Authors:** Chunyu Li, Lei Yue, Xinhong Zhu, Xiaolian Gao, Lin Li, Qingdong Zeng, Fen Yang, Guiyuan Qiao, Qingrong Fu

**Affiliations:** 1 School of Nursing, Hubei University of Chinese Medicine, No. 16, Huangjiahuxi Road, Hongshan District, Wuhan, 430065 Hubei China; 2 Hubei Shizhen Laboratory, Wuhan, 430065 China; 3 Rehabilitation Medicine Center/Tuina Department, Hubei Provincial Hospital of Traditional Chinese Medicine, Wuhan, 430061 China; 4 Affiliated Hospital of Hubei University of Chinese Medicine, Wuhan, 430061 China; 5 Hubei Key Laboratory of Theory and Application Research of Liver and Kidney in Traditional Chinese Medicine (Hubei Province Hospital of Traditional Chinese Medicine), Wuhan, 430061 China; 6 The First Clinical Medical School, Hubei University of Chinese Medicine, Wuhan, 430065 China

**Keywords:** Sleep, Emotion, Behavior, Latent class analysis, Cumulative score, Adolescent

## Abstract

**Background:**

Despite the established association between sleep problems and emotional and behavioral problems in adolescents, there is limited evidence on the relationships of independent, cumulative, and distinct patterns of sleep problems with emotional and behavioral problems.

**Methods:**

Cross-sectional data were collected from middle school students in Daye City, Hubei Province, China, with 1,381 participants included. Emotional and behavioral problems and sleep problems were assessed using the Strengths and Difficulties Questionnaire and Pittsburgh Sleep Quality Index, respectively. Latent class analysis was used to identify the latent patterns of sleep problems, and linear regression analyses were used to explore the associations between sleep problems (including independent types and cumulative scores) and emotional and behavioral problems. The Bolck-Croon-Hagenaars method was applied to compare emotional and behavioral problems across distinct sleep problem patterns. Additionally, relative importance analysis was conducted to estimate the relative contribution of each sleep problem to the variance in emotional and behavioral problems.

**Results:**

Prolonged sleep latency, short sleep duration, inefficient sleep, sleep disturbances, use of sleep medications, and daytime dysfunction were independently correlated with higher levels of emotional and behavioral problems, except for poor subjective sleep quality. Among these sleep problems, daytime dysfunction contributed the most to the variance in emotional (23.5%) and behavioral problems (30.4%). Additionally, the more sleep problems adolescents experienced, the more severe their emotional and behavioral problems were. Three distinct patterns of sleep problems were identified: “global sleep dysfunction”, “disturbed and non-restorative sleep”, and “mild sleep problems”. Adolescents with the “global sleep dysfunction” pattern exhibited the most severe emotional and behavioral problems, whereas those with the “mild sleep problems” pattern exhibited the least severe.

**Conclusions:**

The findings highlight the need to consider the types, numbers, and patterns of sleep problems when developing tailored interventions to alleviate emotional and behavioral problems in adolescents.

**Supplementary Information:**

The online version contains supplementary material available at 10.1186/s13034-026-01104-6.

## Introduction

Adolescence is a critical developmental stage during which adolescents undergo major physical, psychological, social, and interpersonal changes [36]. This transitional phase renders them vulnerable to emotional and behavioral problems (e.g., emotional symptoms, hyperactivity, conduct problems, and peer relationship issues) [16], with an overall prevalence of 19% across all types of such problems [13]. Growing evidence has shown that emotional and behavioral problems in adolescence may compromise long-term mental health [46], and even lead to severe adverse outcomes such as school dropout [28, 29], delinquency [27], and suicide [20]. Without being addressed promptly, these problems are likely to endure and exacerbate the cumulative burden on both society and the economy. Therefore, there is an urgent need to identify risk factors for emotional and behavioral problems in adolescents in order to develop targeted and effective prevention programs.

Sleep problems are highly prevalent among adolescents [41]. Multiple biological, social, psychological, cultural, and familial factors contribute to their emergence ([9]; Sadeh [48]), , manifesting as insufficient sleep duration, poor subjective sleep quality, and daytime dysfunction [35]. Especially in China, limited educational resources and rising educational demands have resulted in fierce enrollment competition [51]. This competition exposes Chinese adolescents to greater academic pressure, potentially increasing their likelihood of sleep problems [35, 57]. Accumulating evidence has demonstrated that sleep problems are strongly associated with emotional and behavioral problems in adolescents [24, 53, 60].

Sleep problems should be regarded as a multidimensional construct [7, 39]. Although existing evidence has established their association with emotional and behavioral problems, most studies have focused only on a single aspect or used a composite score to represent overall sleep problems. Therefore, it is necessary to further investigate this relationship by simultaneously considering multiple aspects of sleep problems. Moreover, most prior studies that explored the relationship between the two relied on regression analysis [24, 52], which failed to account for the multicollinearity of multiple sleep problems [56]. Meanwhile, they have not distinguished which sleep problem is most closely related to emotional and behavioral problems. In contrast, relative weight analysis can eliminate multicollinearity and identify the sleep problems that contribute most significantly to emotional and behavioral problems [25], thereby providing the most prioritized intervention target especially when resources are limited.

In addition, studies have shown that sleep problems do not occur alone but often coexist [26] and have cumulative effects [34, 58]. As proposed by the “cumulative risk model” [14], the accumulation of adverse factors tends to elevate an individual’s risk of disorder. Evidence has shown that cumulative unhealthy sleep habits during adolescence can affect substance use in young adulthood [23]. However, it remains unclear whether cumulative sleep problems are related to adolescents’ emotional and behavioral problems. Drawing on this framework, the present study quantified different **s**leep problems into a cumulative sleep problem score to assess their cumulative effect.

Nevertheless, the cumulative score focuses on the quantity of sleep problems, ignoring their coexistence patterns. Latent class analysis (LCA) is a person-centered method for qualitatively identifying distinct subgroups with shared specific external characteristics [61]. In the field of sleep research, several studies have used LCA to identify distinct patterns of sleep problems among adolescents [50, 55]. However, to our knowledge, the relationship between sleep problem patterns and emotional and behavioral problems has rarely been explored.

In summary, the present study aimed to simultaneously explore the relationships between independent, cumulative, and latent patterns of sleep problems and emotional and behavioral problems among adolescents, thereby providing a more nuanced delineation of this association.

## Methods

### Participants

This study utilized data from a cross-sectional survey conducted among Chinese middle school students. Participants were recruited from three middle schools in Daye City, Hubei Province, taking into account the representativeness of adolescent students in the region, as well as collaboration and convenience. A total of 1,725 adolescents voluntarily participated in the survey. This study excluded 204 participants with missing information on sleep problems and emotional and behavioral problems, as well as 140 participants with either abnormal sleep duration (e.g., getting up after 8:00 a.m. on school days, i.e., after classes have started) or regular responses to the questionnaire on emotional and behavioral problems. A total of 1,381 adolescents were finally included in the study.

### Procedure

After obtaining permission from the principals and head teachers of the target schools, the researchers distributed the questionnaires in the classrooms during the self-study periods on the same day. Prior to the distribution, informed consent was obtained from all adolescent participants and their legal guardians, and the principle of voluntary participation was emphasized. All participating adolescents received a pen of the same style and price as a token of appreciation, which was intended to compensate them for the time and inconvenience involved in the study. The gift was chosen based on the participants’ age and student status, as well as its reasonable value, to avoid undue influence. This survey was approved by the Medical Research Ethics Committee of Hubei University of Chinese Medicine (Reference No.: 2025049).

### Measures

#### Emotional and behavioral problems

The Strengths and Difficulties Questionnaire (SDQ) is a brief 25-item questionnaire used to assess emotional and behavioral problems over the preceding 6 months, rated on a 3-point scale (0 = not true, 1 = somewhat true, and 2 = certainly true) [18]. The SDQ includes 5 subscales (i.e., emotional symptoms, peer relationship issues, conduct problems, hyperactivity-inattention, and prosocial behavior), each consisting of 5 items. Detailed items of the SDQ are presented in Table S1. The score for each subscale is calculated as the sum of its respective items, with a score range of 0 to 10. In this study, the two subscales of emotional symptoms and peer relationship issues were summed to create a total score for emotional problems, and the two subscales of conduct problems and hyperactivity-inattention were summed to create a total score for behavioral problems [40]. Higher total scores indicate more severe emotional or behavioral problems. The questionnaire has been validated for use among Chinese adolescents [65].

#### Sleep problems

Pittsburgh Sleep Quality Index (PSQI), developed in 1989 by Buysse and colleagues, was employed to evaluate individual sleep quality over the past month [8]. The PSQI consists of 19 self-rated items and 5 other-rated items. Among them, 18 self-rated items are further divided into 7 components: subjective sleep quality (item 6), sleep latency (items 2 and 5a), sleep duration (item 4), sleep efficiency (items 1, 3, and 4), sleep disturbances (items 5b-5j), use of sleep medications (item 7), and daytime dysfunction (items 8 and 9). Each component is scored from 0 (no problem) to 3 (severe problem), with higher scores indicating worse sleep quality. For the purpose of this study, each component was dichotomized into two categories: no sleep problem (score = 0) and the presence of a sleep problem (score ≥ 1) [24]. The satisfactory validity and reliability of the PSQI have been demonstrated in a sample of Chinese adolescents [19].

#### Covariates

Covariates, including sociodemographic variables, BMI, pubertal development, and physical activity, were selected based on established evidence that these variables are associated with sleep problems as well as emotional and behavioral problems [5, 12, 43, 47, 54, 63, 69]. Sociodemographic details included adolescent age, gender, family economic status (poor/ordinary/good), and parental education (elementary or lower, middle school, high school, and college or higher).Pubertal development was assessed as a binary measure (started/not started), with “started” indicating that puberty had begun. BMI was calculated as weight in kilograms divided by the square of height in meters. Physical activity was measured using the Physical Activity Ranking Scale-3 **(** [33]**)**, which comprises 3 items evaluating exercise intensity (1 = “light” to 5 = “heavy”), single-session duration (0 = “less than 10 min” to 4 = “over 1 h”), and monthly frequency (1 = “less than once per month” to 5 = “once every day”) in the past month. The total score (ranging from 0 to 100) was calculated as the product of these three item scores. Higher total scores indicate a higher level of physical activity.

### Statistical analysis

Firstly, sample characteristics were summarized using descriptive statistics. Continuous variables were reported as the mean and standard deviation, while categorical variables were presented as frequency and percentage.

Secondly, LCA was conducted to identify potential patterns of sleep problems based on the 7 components of PSQI. The optimal model was selected based on several criteria, including Akaike’s Information Criterion (AIC), Bayesian Information Criterion (BIC), adjusted Bayesian Information Criterion (aBIC), entropy, the Lo-Mendell-Rubin adjusted likelihood ratio test (LMR), the bootstrap likelihood ratio test (BLRT), and class proportions, as well as the parsimony, utility, and substantive interpretability of the models [37, 44]. Lower values of AIC/BIC/aBIC indicate superior fit. Given that the BIC is more reliable than AIC and aBIC, it is given greater weight when these criteria show inconsistency in model selection [45]. Entropy indicates how accurately the individuals are classified and ranges from 0 to 1, with higher values indicating better classification [10]. Significant BLRT and LMR tests indicate that a “K-class-model” is better than a “K-1-class-model” [68]. If the proportion of individuals in a certain category is less than 5%, it indicates that the subgroup tends to be unstable and difficult to replicate in other studies [44]. After identifying the latent patterns of sleep problems, Wald chi-square tests were used to examine the distribution of seven sleep problems across different patterns. If necessary, post hoc analyses were conducted.

Next, linear regression models were employed to examine the independent effects of seven sleep problems on emotional and behavioral problems. The relative importance analysis was conducted to further identify the contribution of each sleep problem to the variance in emotional and behavioral problems, taking into account the multicollinearity among multiple sleep problems. Then, we summed the scores of seven binary sleep problem variables to generate a cumulative score and investigated the association between cumulative sleep problems and emotional and behavioral problems using linear regression models.

Finally, in order to examine differences in emotional and behavioral problems across the latent sleep problem patterns, the Bolck-Croon-Hagenaars (BCH) approach was employed [4]. This approach corrects for classification error in latent class membership by creating weights. It enables adjusted pairwise comparisons of mean differences in distal outcomes using Wald chi-square tests while preserving the stability of the latent class structure [2, 3].

The significance level was set at *α* = 0.05. Robust standard errors were employed in the regression analyses to account for potential heteroscedasticity [62]. *P*-values were adjusted for multiple testing using the Benjamini-Hochberg method to control the false discovery rate, and an adjusted *P*-value < 0.05 was considered statistically significant [6]. The LCA and BCH methods were performed using Mplus (version 8.3). The relative importance analysis was conducted using R (Version 4.4.1), and all the other analyses were carried out using Stata 18.0.

## Results

### Sample characteristics

The characteristics of the samples are presented in Table 1. The sample consisted of 1,381 adolescents, with a mean age of 14.36 ± 0.94 years and a mean BMI of 19.80 ± 3.01. There were slightly more males (52.6%) than females (47.4%), and puberty had commenced in 93.8% of the participants. The adolescents had a moderate level of physical activity. Parental educational level was dominated by middle school (mothers: 50.8%; fathers: 52.5%). The vast majority of adolescents were from families with ordinary economic status. The most commonly self-reported sleep problem in this sample was poor subjective sleep quality (87.1%), followed by daytime dysfunction (84.7%), sleep disturbances (81.5%), prolonged sleep latency (52.6%), inefficient sleep (18.3%), short sleep duration (15.4%), and use of sleep medications (0.5%). Adolescents reported the highest mean score on hyperactivity-inattention (4.28 ± 2.11) and the lowest mean score on peer relationship issues (2.41 ± 1.54). The mean score for behavioral problems (6.84 ± 3.13) was slightly higher than that for emotional problems (6.18 ± 3.04).

**Table 1:** Sample characteristics (*n* = 1381)

Age (years)	*n* (%)/Mean ± SD
	14.36 ± 0.94
Gender	
Male	727 (52.6)
Female	654 (47.4)
BMI	19.80 ± 3.01
Pubertal development	
Started	1296 (93.8)
Not started	89 (6.2)
Maternal education	
Elementary or lower	168 (12.2)
Middle school	702 (50.8)
High school	313 (22.7)
College or higher	198 (14.3)
Paternal education	
Elementary or lower	90 (6.5)
Middle school	725 (52.5)
High school	366 (26.5)
College or higher	200 (14.5)
Family economic status	
Poor	59 (4.3)
Ordinary	1268 (91.8)
Good	54 (3.9)
Physical activity	32.52 ± 23.76
Poor subjective sleep quality	
Yes	1203 (87.1)
No	178 (12.9)
Prolonged sleep latency	
Yes	726 (52.6)
No	655 (47.4)
Short sleep duration	
Yes	213 (15.4)
No	1168 (84.6)
Inefficient sleep	
Yes	252 (18.3)
No	1129 (81.7)
Sleep disturbances	
Yes	1126 (81.5)
No	255 (18.5)
Use of sleep medications	
Yes	7 (0.5)
No	1374 (99.5)
Daytime dysfunction	
Yes	1170 (84.7)
No	211 (15.3)
Emotional symptoms	3.77 ± 2.35
Peer relationship issues	2.41 ± 1.54
Conduct problems	2.56 ± 1.52
Hyperactivity-inattention	4.28 ± 2.11
Emotional problems	6.18 ± 3.04
Behavioral problems	6.84 ± 3.13

BMI, Body Mass Index

### Latent patterns of sleep problems

Table 2 presents the statistical fit indices of one-to four-class models. Compared with the 1- and 2-class models, the 3-class model exhibited lower AIC, BIC, and aBIC, and its entropy was substantially higher than that of the 2-class model. The *P*-values of LMR and BLRT of the 3-class model were also statistically significant. Additionally, relative to the 3-class model, the 2-class model was overly parsimonious and less theoretically informative (see Fig. S1).

**Table 2:** Fit statistics of latent class analysis solutions (*n* = 1381)

No. of classes	AIC	BIC	aBIC	Entropy	LMR (*P*)	BLRT (*P*)	Class proportions
1	8076.119	8112.733	8090.497				
2	7745.895	7824.354	7776.705	0.444	< 0.001	< 0.001	0.492 / 0.508
**3**	**7597.221**	**7717.524**	**7644.462**	**0.740**	**< 0.001**	**< 0.001**	**0.139 / 0.631 / 0.230**
4	7583.589	7745.737	7647.262	0.736	0.001	< 0.001	0.054 / 0.554 / 0.217 / 0.175

AIC, Akaike Information Criterion; BIC, Bayesian Information Criterion; aBIC, adjusted Bayesian Information Criterion; LMR, the Lo-Mendell-Rubin likelihood ratio test; BLRT, the bootstrap likelihood ratio test. Bolded row represents the identified model

Although the 4-class model was suggested by the results of the LMR and BLRT, it exhibited slightly higher BIC and aBIC and slightly lower entropy than the 3-class model. In addition, one class (5.4%) in the 4 - class model might be unstable and not easily replicable, considering its small proportion in the sample. The inclusion of this class did not improve the substantive interpretability of the results but instead increased the model complexity (see Fig. S2). Thus, by jointly considering statistical fit indices, substantive interpretability, and the parsimony of the models, the 3 - class model was selected as the optimal solution.

**Fig. 1:**
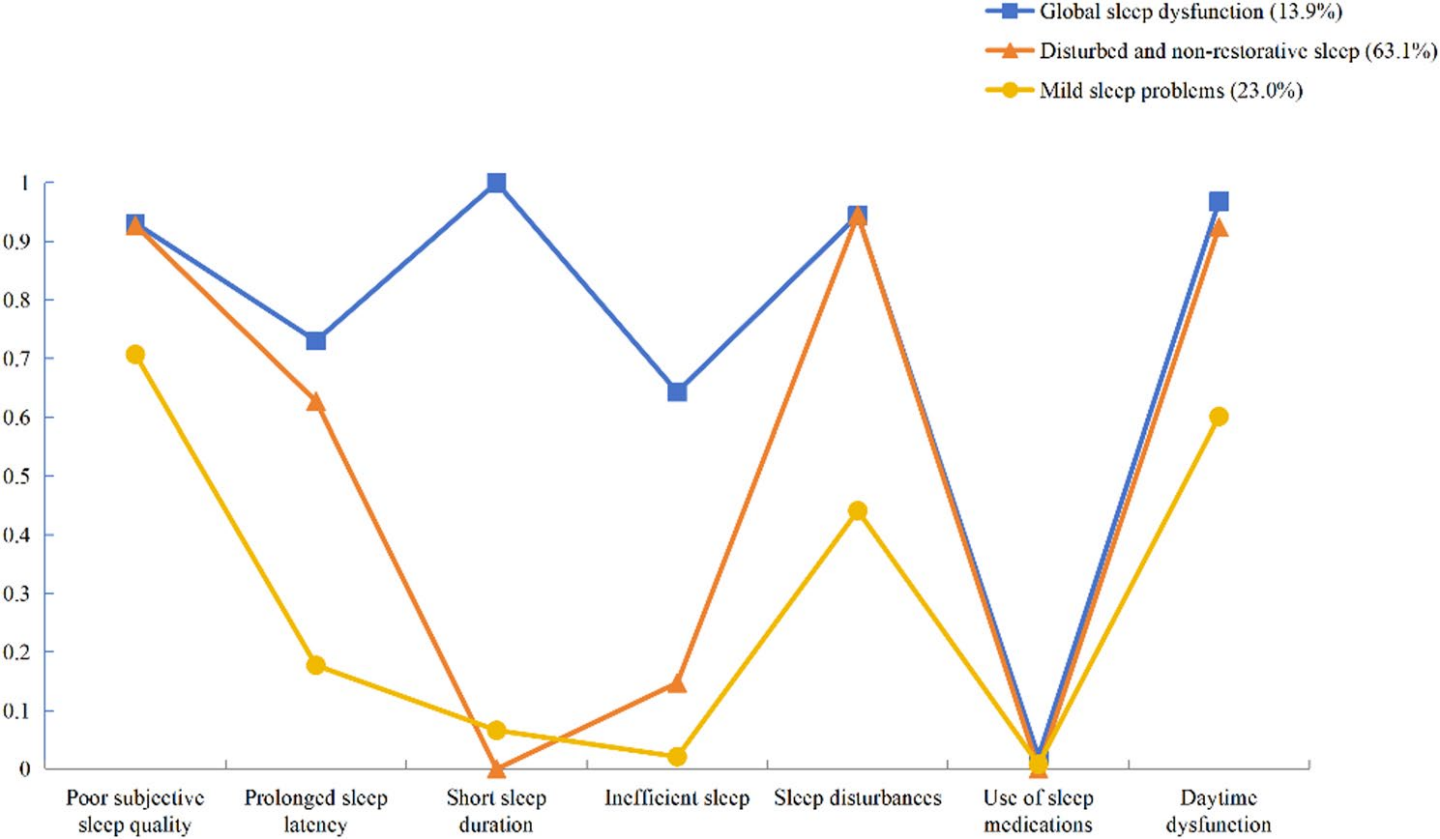
Latent patterns of sleep problems (*n* = 1381). The ordinate represents the probability of the presence of a sleep problem

Based on the 3-class model, Fig. 1 illustrates the probabilities of the 7 components of PSQI in each latent class. The first class of participants (13.9%) exhibited a relatively high probability of multidimensional sleep problems except for the use of sleep medications, and thus, were labeled as “global sleep dysfunction”. Participants in the second class (63.1%) were characterized by high likelihood of poor subjective sleep quality, prolonged sleep latency, sleep disturbances, and daytime dysfunction, and hence, were labeled as “disturbed and non-restorative sleep”. The third class of participants (23.0%) had fewer sleep problems than the other two classes and were therefore labeled as “mild sleep problems”. As shown in Table S2, significant disparities emerged in the prevalence of sleep problems across the three patterns.

### The relationship between independent sleep problems and emotional and behavioral problems

#### Linear regression analysis results

As shown in Table 3, after controlling for age, gender, BMI, pubertal development, maternal education, paternal education, family economic status, and physical activity, other sleep problems were significantly associated with emotional and behavioral problems, except for poor subjective sleep quality. Specifically, prolonged sleep latency (emotional problems: *b* = 0.828, *P* < 0.001; behavioral problems: *b* = 0.775, *P* < 0.001), short sleep duration (emotional problems: *b* = 0.814, *P* = 0.001; behavioral problems: *b* = 0.958, *P* < 0.001), inefficient sleep (emotional problems: *b* = 0.986, *P* < 0.001; behavioral problems: *b* = 1.258, *P* < 0.001), sleep disturbances (emotional problems: *b* = 0.912, *P* < 0.001; behavioral problems: *b* = 1.089, *P* < 0.001), use of sleep medications (emotional problems: *b* = 1.639, *P* = 0.022; behavioral problems: *b* = 3.007, *P* = 0.001), and daytime dysfunction (emotional problems: *b* = 1.187, *P* < 0.001; behavioral problems: *b* = 1.910, *P* < 0.001) were all related to more severe emotional and behavioral problems.

**Table 3:** The relationship between independent and cumulative sleep problems and emotional and behavioral problems (*n* = 1381)

	Emotional problems			Behavioral problems		
	b	se	*P*	b	se	*P*
Independent sleep problems						
Poor subjective sleep quality	-0.173	0.239	0.468	0.064	0.223	0.774
Prolonged sleep latency	0.828	0.155	< 0.001	0.775	0.152	< 0.001
Short sleep duration	0.814	0.244	0.001	0.958	0.249	< 0.001
Inefficient sleep	0.986	0.227	< 0.001	1.258	0.231	< 0.001
Sleep disturbances	0.912	0.190	< 0.001	1.089	0.184	< 0.001
Use of sleep medications	1.639	0.696	0.022	3.007	0.879	0.001
Daytime dysfunction	1.187	0.205	< 0.001	1.910	0.195	< 0.001
Cumulative sleep problems	0.739	0.061	< 0.001	0.982	0.061	< 0.001

All models are adjusted for age, gender, BMI, pubertal development, maternal education, paternal education, family economic status, and physical activity. *se* denotes robust standard errors. *P*-values for independent sleep problems were adjusted for multiple testing using the Benjamini-Hochberg method

#### Relative weight analysis results

Table 4 presents the variance of emotional and behavioral problems explained by each sleep problem. For emotional problems, daytime dysfunction explained the largest proportion of variance (23.5%), followed by sleep disturbances (20.3%), inefficient sleep (20.0%), prolonged sleep latency (19.3%), short sleep duration (16.9%), and use of sleep medications (1.4%). For behavioral problems, the contribution of each sleep problem differed slightly. Daytime dysfunction still accounted for the most variance (30.4%), followed by inefficient sleep (20.4%), sleep disturbances (18.6%), short sleep duration (14.0%), prolonged sleep latency (14.2%), and use of sleep medications (2.4%).

**Table 4:** The raw relative importance and rescaled relative importance of each sleep problem to emotional and behavioral problems (*n* = 1381)

	Emotional problems	Behavioral problems
Prolonged sleep latency	0.031 (19.3%)	0.031 (14.2%)
Short sleep duration	0.026 (16.9%)	0.031 (14.0%)
Inefficient sleep	0.032 (20.0%)	0.045 (20.4%)
Sleep disturbances	0.032 (20.3%)	0.041 (18.6%)
Daytime dysfunction	0.037 (23.5%)	0.067 (30.4%)
Use of sleep medications	0.002 (1.4%)	0.005 (2.4%)

### The relationship between cumulative sleep problems and emotional and behavioral problems

When using cumulative sleep problems as the independent variable, linear regression analysis showed that the greater the number of sleep problems, the more severe the emotional and behavioral problems (emotional problems: *b* = 0.739, *P* < 0.001; behavioral problems: *b* = 0.982, *P* < 0.001). See Table 3.

### Comparison of emotional and behavioral problems across distinct sleep problem patterns

As presented in Tables 5 and 6, after adjusting for age, gender, BMI, pubertal development, maternal education, paternal education, family economic status, and physical activity, statistically significant group differences were observed for both emotional and behavioral problems. Specifically, adolescents with the “global sleep dysfunction” pattern had more severe emotional and behavioral problems than those with the “disturbed and non-restorative sleep” pattern (emotional problems: *χ²* = 25.421, *P* < 0.001; behavioral problems: *χ²* = 33.395, *P* < 0.001) and the “mild sleep problems” pattern (emotional problems: *χ²* = 101.748, *P* < 0.001; behavioral problems: *χ²* = 171.625, *P* < 0.001). Furthermore, adolescents with the “disturbed and non-restorative sleep” pattern showed more severe emotional and behavioral problems than their counterparts with the “mild sleep problems” pattern (emotional problems: *χ²* = 64.561, *P* < 0.001; behavioral problems: *χ²* = 118.152, *P* < 0.001.

**Table 5:** Comparison of emotional problems across distinct sleep problem patterns (*n* = 1381)

	Mean	95% CI	χ²	*P*
Latent patterns of sleep problems				
GSD	8.125	7.533, 8.717		
DNS	6.500	6.271, 6.729		
MSP	4.326	3.905, 4.747		
GSD vs. DNS			25.421	< 0.001
GSD vs. MSP			101.748	< 0.001
DNS vs. MSP			64.561	< 0.001

All models are adjusted for age, gender, BMI, pubertal development, maternal education, paternal education, family economic status, and physical activity. GSD, global sleep dysfunction. MSP, mild sleep problems. DNS, disturbed and non-restorative sleep. *P*-values were adjusted for multiple testing using the Benjamini-Hochberg method

**Table 6:** Comparison of behavioral problems across distinct sleep problem patterns (*n* = 1381)

	Mean	95% CI	χ²	*P*
Latent patterns of sleep problems				
GSD	9.216	8.612, 9.820		
DNS	7.325	7.092, 7.558		
MSP	4.281	3.832, 4.730		
GSD vs. DNS			33.395	< 0.001
GSD vs. MSP			171.625	< 0.001
DNS vs. MSP			118.152	< 0.001

All models are adjusted for age, gender, BMI, pubertal development, maternal education, paternal education, family economic status, and physical activity. GSD, global sleep dysfunction. MSP, mild sleep problems. DNS, disturbed and non-restorative sleep. *P*-values were adjusted for multiple testing using the Benjamini-Hochberg method

## Discussion

We found that among independent sleep problems, daytime dysfunction accounted for the largest proportion of variance in emotional and behavioral problems. A higher number of sleep problems was associated with more severe emotional and behavioral problems. Furthermore, this study identified three distinct patterns of sleep problems in adolescents: “global sleep dysfunction”, “disturbed and non-restorative sleep”, and “mild sleep problems”. Adolescents with the “global sleep dysfunction” pattern exhibited the most severe emotional and behavioral problems, while those with the “mild sleep problems” pattern showed the lowest levels.

In the present study, the high prevalence of sleep problems (particularly poor subjective sleep quality, daytime dysfunction, and sleep disturbances) is similar to that reported in another study that also used the PSQI to screen for sleep problems in adolescents [42]. A likely explanation for the high prevalence may be the changes in the secretion patterns of hormones (such as gonadal hormones and melatonin) during adolescence, which delay the circadian rhythm [21]. The delayed circadian rhythm conflicts with the need for adolescents to wake up early, especially on school days. Hence, it may contribute to sleep disorders and reduced sleep quality, and may leave them sleepy or lacking energy the next day.

Our findings on sleep problem patterns partially align with and extend the limited previous LCA studies on adolescent sleep. Using PSQI-derived sleep features, Xie et al. [64] identified four distinct sleep patterns in Chinese adolescents, namely, “healthy sleepers”, “latency but functional sleepers”, “efficient but dysfunctional sleepers”, and “medicated maladaptive sleepers”. Our “disturbed and non - restorative sleep” pattern (63.1%) shares conceptual similarities with their “efficient but dysfunctional sleepers” pattern (57.5%). Both are characterized by high probabilities of poor subjective sleep quality, prolonged sleep latency, sleep disturbances, and daytime dysfunction. Notably, the additional “latency but functioning sleepers” pattern, characterized by high probabilities of prolonged sleep latency and sleep disturbance but low probabilities of difficulties in other sleep indicators, was not observed in our study. In addition, their “medicated maladaptive sleepers” pattern exhibited abnormalities in all sleep features, whereas our identified patterns did not involve sleep medication use. The discrepancies in findings may be partially attributed to differences in sample characteristics. The participants in our study were junior high school students, while Xie et al. [64] recruited senior high school students. Compared with junior high school students, senior high school students generally experience greater academic pressure, which may exacerbate sleep disturbances and increase the likelihood of using sleep medications [17]. Moreover, a previous study identified three sleep problem patterns in Chinese adolescents using five sleep indicators [67], which also differ from those in our study. Despite common variations in research findings, a small but growing body of LCA research has begun to reveal the inherent heterogeneity in sleep problems [32, 66].

The three latent classes identified in our study reflect qualitatively distinct sleep problem patterns rather than a simple severity gradient. They may have important implications for personalized and targeted intervention. For adolescents with the “disturbed and non-restorative sleep” pattern, cognitive-behavioral therapy for insomnia may be particularly beneficial given their specific sleep characteristics [15, 38]. In contrast, adolescents with the “global sleep dysfunction” pattern may require more comprehensive and multicomponent interventions. Although adolescents with the “mild sleep problems” pattern experienced relatively mild sleep difficulties, early screening and interventions targeting their prominent issues are still needed to prevent progression to more severe sleep problems. A previous study has shown that different subgroups of adolescents at risk for insomnia exhibit varying responses to an insomnia prevention program [11], further supporting the utility of sleep pattern classification for personalized intervention.

Consistent with previous literature [52], sleep problems were associated with more severe emotional and behavioral problems in adolescents. Sleep problems are correlated with an increased vulnerability of the prefrontal cortex [1]. Its core function, including decision-making, reward processing, social interaction, and emotion regulation, may be suppressed, potentially contributing to emotional and behavioral problems. It is worth noting that poor subjective sleep quality showed no statistically significant association with emotional and behavioral problems after controlling for the other six types of sleep problems. Subjective sleep quality is inherently non-specific and vague [66], and other sleep problems may potentially load on the poor subjective sleep quality component [22]. Therefore, acting as a non-specific indicator of overall sleep health, its association with psychopathology may be explained by other more concrete sleep problems.

Relative importance analysis further showed that daytime dysfunction contributed the most to the variance in emotional and behavioral problems. Daytime dysfunction may be associated with adolescents’ reduced ability to focus on classroom content. At the same time, individuals with daytime dysfunction often experience more anxiety and discomfort in social interactions, which may contribute to social exclusion and problems with emotional regulation [59]. This finding underscores the urgent need for interventions aimed at mitigating daytime dysfunction for adolescents with severe emotional and behavioral problems, particularly in resource-limited settings.

Cumulative effect analysis showed that an increase in the number of sleep problems was associated with more severe emotional and behavioral problems. This finding supported the cumulative risk theory [49], which posits that an increase in exposure to risk factors raises the probability of adverse outcomes. Even after accounting for population heterogeneity using a person-centered approach, this association remained significant. The results may indicate not only that sleep problems are independently associated with more severe emotional and behavioral problems, but also that the number and patterns of concurrent sleep problems are key considerations. Therefore, adolescent sleep problems should be given due attention, and timely measures should be taken to prevent the accumulation and escalation of sleep problems.

Several limitations of the present study merit attention. First, given its cross-sectional design, no causal inferences can be drawn from the observed associations. Future longitudinal studies are thus required to validate and expand on these findings. Second, all study information was self-reported by adolescents, which may introduce reporting and recall bias. Previous evidence has demonstrated the good reliability and validity of the PSQI, as well as the correlation between subjective and objective sleep assessment measures [30, 31]. Nonetheless, future studies are warranted to adopt objective sleep measurement tools (e.g., actigraphy devices) to replicate our findings. Third, the participants were conveniently recruited, which limits the representativeness of the sample and the generalizability of the study findings. Future research should replicate these results using more representative samples. Finally, the current analysis did not account for other potential confounding variables, highlighting the need for future research to incorporate a more comprehensive set of covariates.

## Conclusions

This study contributes to the growing literature by comprehensively analyzing independent, cumulative, and latent patterns of sleep problems in relation to adolescent emotional and behavioral problems. Daytime dysfunction emerged as the most prominent independent sleep problem, and a cumulative effect was observed. Regarding distinct sleep patterns, adolescents in the “global sleep dysfunction” pattern exhibited the most severe emotional and behavioral problems. The findings highlight the importance of prioritizing improvements in daytime dysfunction to alleviate emotional and behavioral problems in adolescents. Additionally, tailored interventions are warranted to mitigate and eliminate cumulative and co-occurring sleep problems to promote adolescent emotional and behavioral health.

## Supplementary Information

Below is the link to the electronic supplementary material.

Supplementary Material 1.

## Data Availability

The datasets generated and/or analyzed during the current study are not publicly available but are available from the corresponding author on reasonable request.
