# Supplementary material for: Sleep problems and emotional and behavioral problems among adolescents: examining independent types, cumulative scores, and latent patterns
Source: Child Adolesc Psychiatry Ment Health. 2026 Jun 3;20:100. doi: 10.1186/s13034-026-01104-6 (PMC13450539; doi:10.1186/s13034-026-01104-6)
Supplement: Supplementary file 1 [file 13034_2026_1104_MOESM1_ESM.docx]

**Table S1** The Strengths and Difficulties Questionnaire

| Subscale | Item |
| --- | --- |
| Emotional symptoms | 1. Often complains of headaches, stomach-aches or sickness |
|  | 2. Many worries, often seems worried |
|  | 3. Often unhappy, down-hearted or tearful |
|  | 4. Nervous or clingy in new situations, easily loses confidence |
|  | 5. Many fears, easily scared |
| Peer relationship issues | 6. Rather solitary, tends to play alone |
|  | 7. Has at least one good friend |
|  | 8. Generally liked by other children |
|  | 9. Picked on or bullied by other children |
|  | 10. Gets on better with adults than with other children |
| Conduct problems | 11. Often has temper tantrums or hot tempers |
|  | 12. Generally obedient, usually does what adults request |
|  | 13. Often fights with other children or bullies them |
|  | 14. Often lies or cheats |
|  | 15. Steals from home, school or elsewhere |
| Hyperactivity-inattention | 16. Restless, overactive, cannot stay still for long |
|  | 17. Constantly fidgeting or squirming |
|  | 18. Easily distracted, concentration wanders |
|  | 19. Thinks things out before acting |
|  | 20. Sees tasks through to the end, good attention span |
| Prosocial behavior | 21. Considerate of other people’s feelings |
|  | 22. Shares readily with other children (treats, toys, pencils etc.) |
|  | 23. Helpful if someone is hurt, upset or feeling ill |
|  | 24. Kind to younger children |
|  | 25. Often volunteers to help others (parents, teachers, other children) |

*Note*. Items 7, 8, 12, 19 and 20 are reverse-scored. Except for the five items for prosocial behavior, the remaining 20 items were used in this study.

**Fig.S1** Latent patterns of sleep problems for the 2-class model (*n* = 1381)

*Note*. The ordinate represents the probability of the presence of a sleep problem.

**Fig.S2** Latent patterns of sleep problems for the 4-class model (*n* = 1381)

*Note*. The ordinate represents the probability of the presence of a sleep problem.

**Table S2** Distribution of sleep problems by distinct patterns of sleep problems (*n* = 1381).

|  | GSD | DNS | MSP | *χ²* | *P* | Post-hoc analysis |
| --- | --- | --- | --- | --- | --- | --- |
| Poor subjective sleep quality |  |  |  | 276.376 | < 0.001 | GSD = DNS > MSP |
| Yes | 179 (93.2%) | 834 (95.7%) | 190 (59.7%) |  |  |  |
| No | 13 (6.8%) | 37 (4.3%) | 128 (40.3%) |  |  |  |
| Prolonged sleep latency |  |  |  | 304.857 | < 0.001 | GSD > DNS > MSP |
| Yes | 140 (72.9%) | 554 (63.6%) | 32 (10.1%) |  |  |  |
| No | 52 (27.1%) | 317 (36.4%) | 286 (89.9%) |  |  |  |
| Short sleep duration |  |  |  | 1230.650 | < 0.001 | GSD > MSP > DNS |
| Yes | 192 (100.0%) | 0 (0.0%) | 21 (6.6%) |  |  |  |
| No | 0 (0.0%) | 871 (100.0%) | 297 (93.4%) |  |  |  |
| Inefficient sleep |  |  |  | 335.724 | < 0.001 | GSD > DNS > MSP |
| Yes | 122 (63.5%) | 127 (14.6%) | 3 (0.9%) |  |  |  |
| No | 70 (36.5%) | 744 (85.4%) | 315 (99.1%) |  |  |  |
| Sleep disturbances |  |  |  | 541.681 | < 0.001 | GSD = DNS > MSP |
| Yes | 183 (95.3%) | 825 (94.7%) | 118 (37.1%) |  |  |  |
| No | 9 (4.7%) | 46 (5.3%) | 200 (62.9%) |  |  |  |
| Use of sleep medications |  |  |  | 15.101 | < 0.001 | GSD = MSP > DNS |
| Yes | 4 (2.1%) | 0 (0.0%) | 3 (0.9%) |  |  |  |
| No | 188 (97.9%) | 871 (100.0%) | 315 (99.1%) |  |  |  |
| Daytime dysfunction |  |  |  | 385.713 | < 0.001 | GSD = DNS > MSP |
| Yes | 187 (97.4%) | 824 (94.6%) | 159 (50.0%) |  |  |  |
| No | 5 (2.6%) | 47 (5.4%) | 159 (50.0%) |  |  |  |

*Note*. GSD, global sleep dysfunction. MSP, mild sleep problems. DNS, disturbed and non-restorative sleep. *P*-values were adjusted for multiple comparisons using the Benjamini-Hochberg method.
